# PANoptosis in intestinal epithelium: its significance in inflammatory bowel disease and a potential novel therapeutic target for natural products

**DOI:** 10.3389/fimmu.2024.1507065

**Published:** 2025-01-07

**Authors:** Chuanxiang Zhao, Shan Lin

**Affiliations:** Institute of Medical Genetics and Reproductive Immunity, School of Medical Science and Laboratory Medicine, Jiangsu College of Nursing, Huai’an, China

**Keywords:** inflammatory bowel disease, intestinal epithelium, cell death, PANoptosis, natural products

## Abstract

The intestinal epithelium, beyond its role in absorption and digestion, serves as a critical protective mechanical barrier that delineates the luminal contents and the gut microbiota from the lamina propria within resident mucosal immune cells to maintain intestinal homeostasis. The barrier is manifested as a contiguous monolayer of specialized intestinal epithelial cells (IEC), interconnected through tight junctions (TJs). The integrity of this epithelial barrier is of paramount. Consequently, excessive IEC death advances intestinal permeability and as a consequence thereof the translocation of bacteria into the lamina propria, subsequently triggering an inflammatory response, which underpins the clinical disease trajectory of inflammatory bowel disease (IBD). A burgeoning body of evidence illustrates a landscape where IEC undergoes several the model of programmed cell death (PCD) in the pathophysiology and pathogenesis of IBD. Apoptosis, necroptosis, and pyroptosis represent the principal modalities of PCD with intricate specific pathways and molecules. Ample evidence has revealed substantial mechanistic convergence and intricate crosstalk among these three aforementioned forms of cell death, expanding the conceptualization of PANoptosis orchestrated by the PNAoptosome complex. This review provides a concise overview of the molecular mechanisms of apoptosis, necroptosis, and pyroptosis. Furthermore, based on the crosstalk between three cell deaths in IEC, this review details the current knowledge regarding PANoptosis in IEC and its regulation by natural products. Our objective is to broaden the comprehension of innovative molecular mechanisms underlying the pathogenesis of IBD and to furnish a foundation for developing more natural drugs in the treatment of IBD, benefiting both clinical practitioners and research workers.

## 1 Introduction

Inflammatory bowel disease (IBD) is a chronic idiopathic inflammation disease of the gastrointestinal tract, encompassing ulcerative colitis (UC) and Crohn’s disease (CD) (1). Characterized by inflammation and recurrent ulceration, UC dominates the colonic mucosa, whereas CD manifests any parts of the entire gastrointestinal tract (2). A global epidemiological survey has demonstrated the rise prevalence not only in Western nations but also in emerging countries (3). Despite the ongoing ambiguity surrounding its etiology and pathogenesis, recent studies have elucidated that the intricate interplay between genetic, environmental and immune factors are indispensable trigger for progression to intestinal epithelial barrier damage (4). Consequently, the concept of “mucosal healing” that necessitate the complete regeneration of the intestinal mucosa has been raised as the therapeutic benchmark of IBD (5, 6). Mounting evidence underscores that mismanaged intestinal epithelial cells (IEC) death compromised barrier breach, which underlies instances of widespread epithelial erosion (7, 8). So undoubtedly, a more profound grasp of the IEC cell death paradigm is imperative.

Apoptosis, necroptosis, and pyroptosis are extensively studied forms of cellular demise, each featuring unique morphological and biochemical changes. These processes are meticulously choreographed by tightly-structured signaling cascades of reactions and molecules in response to a certain signal or stimuli, aiming at eliminating unwanted or damaged cells to maintain tissue homeostasis (9). Apoptosis is a non-lytic cell death with an integral cellular membrane and is considered immunologically silent. In contrast, necroptosis and pyroptosis are lytic and inflammatory form of unregulated and accidental cell death (9). Historically, it has been viewed that apoptosis, necroptosis, and pyroptosis act in parallel without overlap, but the three PCDs have recently shown to be tightly interconnected and interact with each other, laying a theoretical foundation for a novel form of PCD known as PANoptosis (10, 11). Comprehensive research has demonstrated abnormal apoptosis, necroptosis, and pyroptosis of IEC during the onset and progression of IBD as well as the complex crosstalk among them (7). Therefore, PANoptosis may represent an innovative therapeutic target for the effective treatment of IBD.

In this review, we present a concise summary of apoptosis, necroptosis, and pyroptosis, subsequently introducing a more elaborate understanding about the intricate interplay among them within IEC to investigate their potential relationship with IBD. Building on this foundation, we further detail the recent advance of PANoptosis in IEC and its regulation by natural products. We aims to offer theoretical basis and reference for targeting PANoptosis in IEC, thereby fostering the development of more effective therapeutic regimens and pharmacological interventions to improve the efficacy of IBD therapy in clinical practice.

## 2 The overview of apoptosis, necroptosis, and pyroptosis

### 2.1 Apoptosis

Apoptosis, the first discovered form of programmed cell death, is a physiological and proactive “conscious suicide” behavior (12). Under specific physiological or pathological conditions, apoptosis is initiated through either receptor-mediated (extrinsic) or mitochondria (intrinsic) pathways, marked initially by the cellular shrinkage and rounding, nuclear fragmentation and chromatin condensation (12). Subsequently, the apoptotic cell features the plasma membrane blistering, tightly encapsulating the cellular debris, culminating in forming the apoptotic bodies. Apoptotic bodies are engulfed by adjacent parenchymal cells and macrophages, and thus this process does not elicit an inflammation response in surrounding tissues (9, 12).

The extrinsic apoptotic pathway is initiated by the binding of extracellular death ligands (TNF family members or Fas ligand) to their corresponding death receptors (DR) on the plasma membrane. Following, the cytoplasmic death domain of death receptors recruits adapter proteins (FADD or TRADD), and then the precursor of caspase-8 is recruited to form the death-inducing signaling complex (DISC), activating the initiator caspase-8 and further activating effector caspases-3/7, ultimately leading to apoptosis (9). The intrinsic (mitochondria) apoptotic pathway is induced by internal apoptotic stimuli, such as oxidative stress, hypoxia, toxic substances, or cytokine deprivation. These stimuli cause the B-cell lymphoma-2 (Bcl-2) protein family to alter the permeability of the mitochondrial membrane, releasing cytochrome c within the mitochondria into the cytoplasm. Subsequently, cytochrome c binds to apoptotic protease activating factor-1 (Apaf-1), facilitating apoptosome assembly, which ignites pro-caspase-9 (13, 14). Then, the activated initiator caspase-9 further activates the effector proteins caspase-3/7, amplifying downstream signals and culminating in apoptosis (14).

### 2.2 Necroptosis

Necroptosis is a lytic and inflammatory form of PCD independent of caspases, typically occurring when pathogens or chemical mediators inhibit apoptosis (15). Morphologically, necroptotic cells feature with necrotic cells, including swollen mitochondria, the explosive rupture of plasma membrane and cell lysis with the leakage of cytosolic constituent into the surrounding tissues (9, 15).

Caspase-8 determines whether the cell undergoes apoptosis or necroptosis. When caspase-8 is inactivated or inhibited by pathogens or chemical mediators, the activated RIPK3-mediated necrosome is formed (16). Primarily, the external stimulus (such as TNF-α, Fas ligand and TLR ligands) binds to death receptors (such as TNFR1 and Fas) and pattern recognition receptors (PRRs, such as Toll-like receptor), which then recruits and activates receptor-interacting kinase 1 (RIPK1). Following, the activated RIPK1 recruits and phosphorylates receptor-interacting kinase 3 (RIPK3) to form the RIPK1-RIPK3 complex (necrosome), then recruiting and phosphorylating mixed lineage kinase domain-like (MLKL) (9). The phosphorylated MLKL translocates to cellular membranes and lyses the cell by forming membrane pores (17). The consequent membrane rupture results in the release of DAMPs, inevitably triggering an inflammation response.

### 2.3 Pyroptosis

Pyroptosis is a lytic and inflammatory form of PCD dependent of a series of caspase families to induce the assembly and activation of inflammasome in response to bacterial or pathogen infections (18). Pyroptotic cells exhibit the distinct and characterized morphology with cell swelling, DNA fragmentation within the intact nucleus and plasma membrane rupture, ultimately leading to cell lysis with the release of inflammatory factors (9).

Pyroptosis is induced by two primary mechanisms: the canonical (caspase-1 dependent inflammasome activation) and the non-canonical (caspase-1 independent inflammasome activation) pathways. In the canonical pathway, pattern recognition receptors (PRR), such as TLRs and Nod-like receptors (NLRs), sense pathogen-associated molecular patterns (PAMPs) or damage-associated molecular patterns (DAMPs) to initiate inflammasome sensors (19). The sensors generally comprise Nod-like receptor protein 3 (NLRP3), NLR family pyrin domain-containing 1 (NLRP1), NLR family CARD domain containing 4 (NLRC4), absent in melanoma 2 (AIM2), and pyrin proteins, with NLRP3 being the most extensively studied. The activated inflammasome sensor then enlists the adapter protein apoptosis-related speck-like protein (ASC) and pro-caspase-1 to form inflammasome (9). Then, pro-caspase-1 is hydrolyzed and converted into the catalytically active form caspase-1, which further cleaves gasdermin D (GSDMD), pro-IL-1β and pro-IL-18. The processed GSDMD releases the N-terminal fragment of GSDMD (GSDMD-N), which inserts into the cell membrane to form pores to leaking the mature IL-1β and IL-18 as well as other DAMPs, thereby amplify the inflammatory response (20).

The non-canonical pathway is launched by lipopolysaccharide (LPS) from gram-negative bacterial. LPS directly interacts with and activates human caspase-4/5 and its murine ortholog caspase-11 to cleave GSDMD, thus inducing inflammation (21, 22). Beyond forming membrane pores, GSDMD-N also facilitates the activation of the non-canonical NLRP3 inflammasome and caspase-1, which cleaves pro-IL-1β and pro-IL-18 in a cell-intrinsic manner (23). With in-depth investigates, the caspase-3/8-dependent pyroptotic pathway and the granzyme-mediated GSDMD- independent pyroptosis pathway have recently been revealed (24). When cell is treated with partial chemical inducers, pyroptosis is induced by caspase-3-mediated cleavage of GSDME, yielding a GSDME-N fragment with the pore-forming activity, while caspase-8 specifically cleaves GSDMC to trigger cell death pathway (24, 25). In addition, granzyme A/B involves in extensive pyroptosis by cleaving GSDMB and GSDME, respectively (26, 27).

## 3 The crosstalk among apoptosis, necroptosis, and pyroptosis in IEC involving in IBD

The appropriate model of IEC death is crucial for maintaining intestine homeostasis. However, excessive and abnormal IEC death programmers can have catastrophic consequences, such as the onset of inflammatory bowel diseases. The epithelium of patients with UC or CD manifests high level of cell deaths (7, 8). Substantial evidence have well-established the involvement of apoptosis, necroptosis, and pyroptosis of IEC in the onset of IBD. Simultaneously, the three models of cell death described above are interconnected and superimposed at multiple levels, which have been mostly described as ‘crosstalk’. Caspase-8 and its adapter FADD are the core molecular bridging apoptosis, necroptosis and pyroptosis in IEC (7). In response to TNF and TLR, caspase-8 is activated, which not only ignites the downstream executioner caspase-3/7 in the apoptotic pathway, but also cleaves RIPK1 and RIPK3 in the necroptotic pathway (28–31). Additionally, caspase-8 and FADD process caspase-1, inflammasome assembly, and GSDMD activation to launch pyroptosis (31–33).

**Figure 1:**
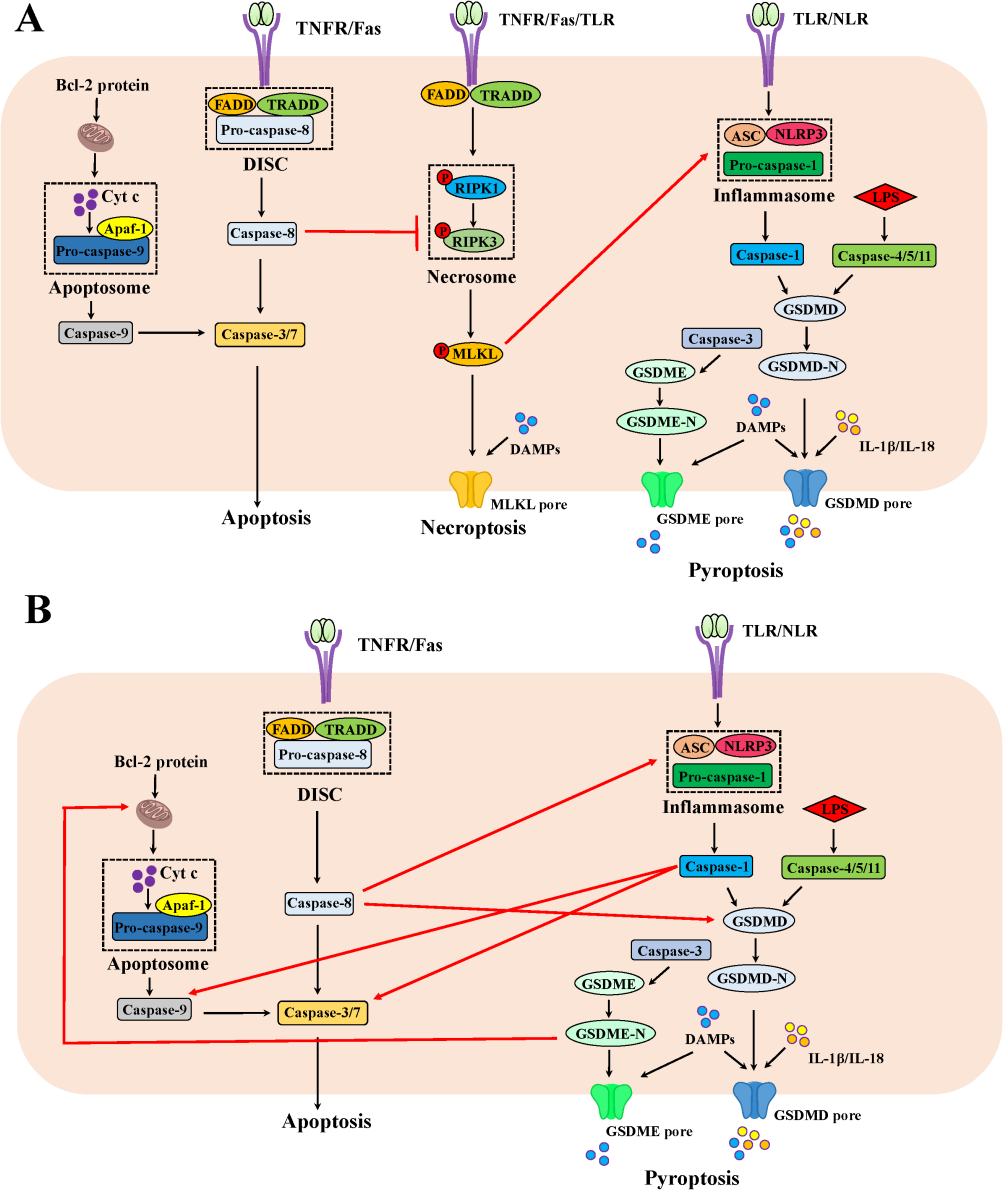
The mechanism of apoptosis, necroptosis and pyroptosis as well as the crosstalk among them in IEC. **(A)** The crosstalk between apoptosis and necroptosis as well as necroptosis and pyroptosis in IEC. Beyond cleaving the effector caspases-3/7 in the apoptotic pathway, the apoptotic molecule caspase-8 could cleave RIPK1, thus inhibiting necroptosis. In addition, the phosphorylated MLKL could foster ASC polymerization and following lead to caspase-1 activation to trigger pyroptosis. **(B)** The crosstalk between apoptosis and pyroptosis in IEC. The apoptotic molecule caspase-8 could process GSDMD activation and interact with ASC to launch pyroptosis. The apoptotic effector caspase-3 is indispensable for GSDME-mediated pyroptosis. When GSDMD is absent, the pyroptotic molecule caspase-1 proceeds to activate caspase-3/7 and caspase-9. The pyroptotic molecule GSDME-N fragment permeabilizes the mitochondrial membrane to induce cytochrome c release and ensuing activate the apoptosome, fueling the mitochondrial apoptotic pathway.

Caspase-8 switches apoptosis and necroptosis (**Figure 1A**). An early study demonstrated that TLR stimulation induced apoptosis and increased shedding of IEC under inflammatory conditions, which was related to the activated caspase-8 (34). The activated caspase-8 also processed gasdermin-D-mediated pyroptosis-like death of epithelial cells and ensuing ileitis (31). In situation where caspase-1 was deleted in IEC, the inflammasome sensors NLRP1b and NLRC4 triggered apoptosis by ASC-dependent caspase 8 activation (35). Under steady state conditions, caspase-8 curbs RIPK1 and RIPK3 activity by proteolytic cleavage in IEC, thereby impeding necroptosis (31). Thus, IEC lacking caspase-8 or FADD due to epithelial cell-specific deletion underwent RIPK3-dependent necroptosis instead of apoptosis in response to TLR or TNF stimulation, leading to a complete absence of Paneth cell, serious tissue damage, enteritis and severe erosive colitis *in vivo* (36–38). Similarly, the deficiency of intestinal epithelial caspase-8 signaling induced necroptosis-mediated enteritis and high lethality after *Salmonella* Typhimurium infection (39). Regarding RIPK1, mice suffering from the deficiencies in both RIPK1 and FADD in IECs displayed RIPK3-dependent IEC necroptosis, Paneth cell loss and focal erosive inflammatory lesions in the colon (40). In line with the results observed in mice, patients with a biallelic 710A > G mutation in the caspase-8 gene presented the increased necroptosis instead of apoptosis in the gut with non-resolving inflammation (41). The imaging *in vivo* showed that IEC necroptosis is considered the basis for the micro erosions and epithelial gaps observed in mice and humans, which is consistent with the significantly high expression of RIPK3 in the terminal ileum of CD patients (38, 42). Indeed, the specific cell type of IEC necroptosis may be Paneth cell. Paneth cells in humans and mice represented a high level of RIPK3 expression (38). Interestingly, Paneth cells without caspase-8 do develop but undergo necroptosis *in vivo* (38). As is well-known, Paneth cells are predominantly rich in the ileum. This may provide an explanation for why mice deficient in caspase-8 bear ileitis but no colitis (38). So far, however, no study has illustrated RIPK3 as a susceptibility gene for IBD in genetic research. This evidence hints that necroptosis of Paneth cells is not a causative factor, but rather a contributing factor supporting intestinal inflammation. In a word, necroptosis is frequently encountered in Paneth cells when apoptosis fails to be induced due to caspase-8 deficiency in IEC.

Although blocking apoptosis through restricting caspase-8 in IEC and switching their death to necroptosis cause overt intestinal inflammation, it may be a self-protection mechanism evolved by the body when the caspase-8-dependent apoptotic pathway has been hijacked by microbes or viral infection. CrmA from the cowpox virus or B13R from the vaccinia virus encodes caspase-8 inhibitor, thus preventing infected cells from apoptosis; however, RIPK3-dependent necroptosis serves as a backup mechanism for killing infected cells and ensures host survival (43, 44). Nevertheless, some pathogens prevent epithelial cell death by delivering effectors via the type III secretion system to inhibit both apoptosis and necroptosis, which can maintain their replicative niche and multiply within cells. For example, *Shigella flexneri* hijacked colonic epithelium via a dual mechanism: it yielded OspC1 to impede caspase-8, thus blocking apoptosis; simultaneously, it also yielded OspD3 to degrade RIPK1 and RIPK3, thereby preventing necroptosis (45).

Collectively, the afore-mentioned studies hint that co-inhibition of caspase-8 and RIPK3 seemingly protects IEC from cell death. Bewilderingly, mice with deletion of RIPK3 with the FLIP proteins-induced caspase-8 inhibition in IEC protected from neither cell death nor inflammation (46). However, the co-ablation of caspase-8 and MLKL downstream of RIPK3 or FADD and RIPK3 in IEC fully protected against cell death and prevented ileitis in mice (31). These evidence imply the involvement of additional molecules in inducing cell apoptosis or necroptosis beyond caspase-8 or RIPK3, respectively. Alternatively, the involvement of another form of cell death in this process might provide another explanation for this observation.

Although no knowledge about additional molecules to induce cell necroptosis independent of RIPK3 has been established, some evidence point out the regulation of pyroptotic molecules on apoptosis beyond caspase-8 (**Figure 1B**). For instances, the canonical molecule of pyroptosis, caspase-1, could cleave the conventional aspartate activation sites (Asp^23^ and Asp^198^) of caspase-7 in macrophages in response to *Salmonella* Typhimurium infection combined with LPS and ATP (47). When GSDMD is absent, caspase-1 also proceeds to activate caspase-3 and caspase-9 as well as trigger mitochondrial damage in macrophages, thereby triggering the apoptotic signaling (48). In addition, GSDMD-N and GSDME-N fragments permeabilized the mitochondrial membrane to induce cytochrome c release and ensuing activate the apoptosome in HEK293T cells, fueling the mitochondrial apoptotic pathway (49). Further studies are required to address whether the regulation of pyroptotic molecules on apoptosis also exists in IEC and whether they affect IBD pathogenesis.

As for the involvement of another cell death, mounting evidence now point out significant contributions of pyroptosis in IECs to the onset of IBD, which could be regulated by apoptotic or necroptotic molecules (**Figure 1**). Elevated levels of GSDMB, GSDMD and GSDME were obviously detected in the inflamed colonic mucosa of IBD patients, predominantly localizing to the intestinal mucosal epithelium (50–52). The apoptotic effector caspase-3 is indispensable for TNBS-induced and GSDME-mediated pyroptosis in IEC (52). Studies with mice carrying enzymatically inactive caspase-8 revealed that the CASP8-RIPK1 platform shared by apoptosis and necroptosis was genetically associated with ASC, the adaptor protein of inflammasomes. The DED domain of caspase-8 in cells from these mice interacted with ASC, triggering pyroptosis and severe inflammation in the intestine (53, 54). Additionally, knockdown of caspase-8 or inhibition of its function promoted RIPK3-mediated inflammasome NLRP3 activation in macrophage, independent of MLKL (55). Similarly, MLKL could foster ASC polymerization and following lead to caspase-1 activation in response to combined treatment of a TLR3 agonist poly(I:C) and zVAD in macrophage (56). The effector molecule of necroptosis MLKL activated NLRP3 inflammasome (57, 58). Therefore, the IEC death in mice with co-ablating RIPK3 and caspase-8 could be rescued when the pyroptotic mediator was deleted (53, 54).

In summary, the crosstalk of events underlying apoptosis, necroptosis and pyroptosis in IEC can be succinctly outlined as follows: caspase-8 switches apoptosis and necroptosis in IEC (**Figure 1**). TLR or TNF immoderate stimulation induced the activation of caspase-8 in IEC, thus initiating an apoptotic fate. Concurrently, caspase-8 fosters gasdermin-D-mediated pyroptosis-like death of epithelial cells. When caspase-8 is deleted or dysfunctional due to pathogen-mediated or pharmacological inhibition, caspase-8 mediated-apoptosis and pyroptosis could not be triggered, resulting in RIPK3-mediated necroptosis of IEC or additional molecules igniting apoptotic/pyroptosis signals in IEC. For example, caspase-1, GSDMD and GSDME induced apoptosis, while caspase-3 or MLKL orchestrate pyroptosis. These excessive cell death modalities, based on the crosstalk of molecules, collectively cause catastrophic intestinal inflammation.

## 4 The role of PANoptosis in IEC and its regulation by nature products

### 4.1 The role of PANoptosis in IEC

Based on the extensive cross-talk between PCD pathways, the conceptualization of a united cell death modality, named PANoptosis, was proposed in 2019 (59). PANoptosis (“P”, pyroptosis; “A”, apoptosis; “N”, necroptosis) is induced by multifaceted PANoptosome complexes with key features of pyroptosis, apoptosis, and/or necroptosis, which could not be fully accounted for by any other PCD pathway alone. The PANoptosome components are tripartite, consisting of: 1) PAMPs or DAMPs sensors like ZBP1, AIM2, and NLRP3; 2) scaffolding proteins such as ASC and FADD; 3) catalytic effectors including RIPK1, RIPK3, CASP-1 and CASP-8 (59). To date, several distinct PANoptosome complexes have been identified, featuring unique sensors and regulators, such as the ZBP1-, AIM2-, RIPK1-, and NLRP12-PANoptosomes (10).

**Figure 2:**
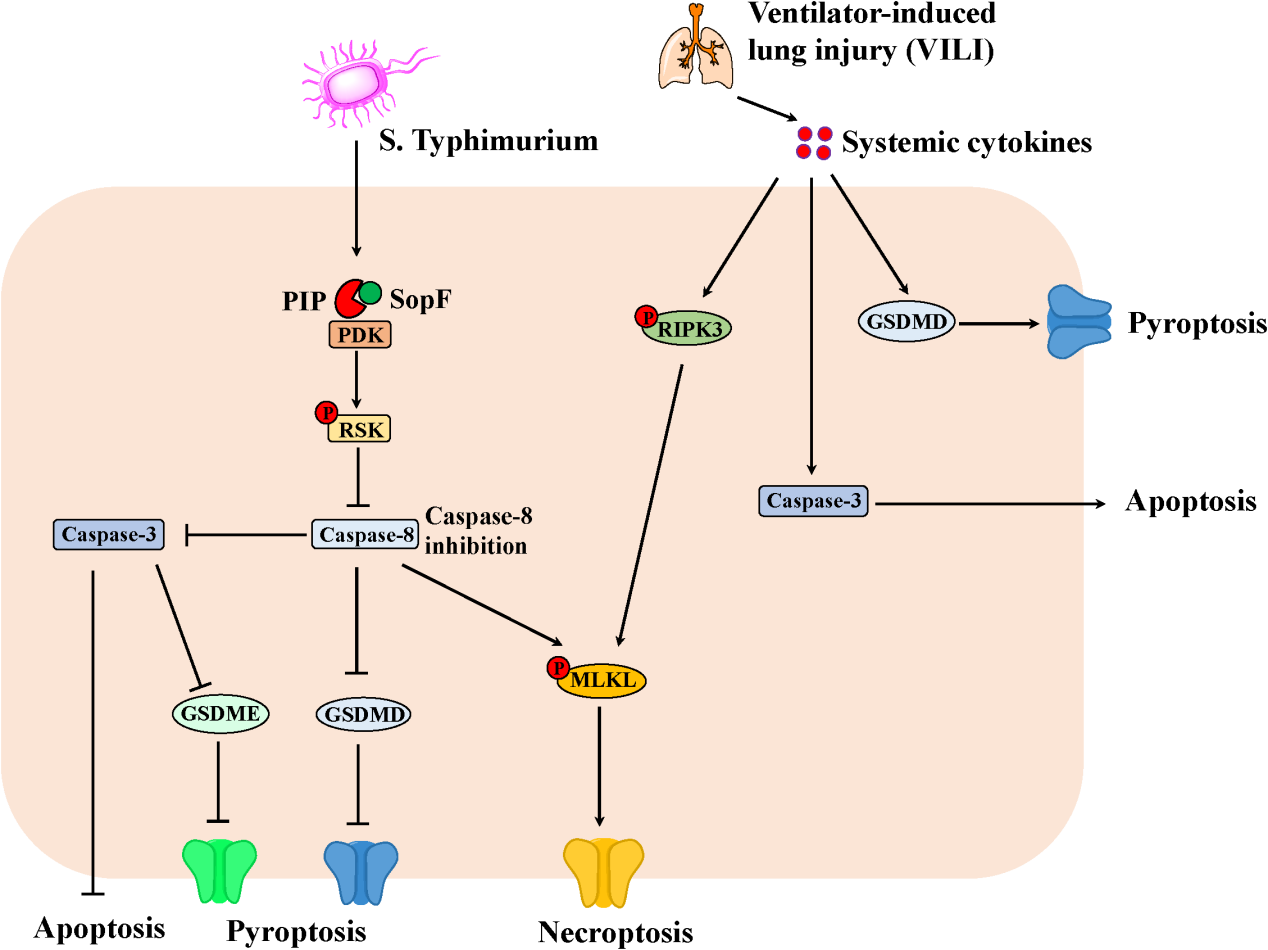
The PANoptosis in IEC involving in the intestinal inflammation. *S. Typhimurium* effector SopF inactivates caspase-8 through the PDK1-RSK signaling, thereby inhibiting apoptosis and pyroptosis of IEC with the promotion of necroptosis. Consequently, SopF attenuates intestinal inflammation, however, promotes bacterial dissemination, which exacerbates systemic infection. Moreover, ventilator-induced lung injury (VILI) results in systemic cytokines, which promotes the expression of Caspase-3, N-GSDMD and p-RIPK3 in the gut. Thus, apoptosis, necroptosis and pyroptosis can lead to intestinal injury and inflammation in mice.

Given the crosstalk of apoptosis, pyroptosis and necroptosis in IEC, the researches has demonstrated the important regulator of PANoptosis in the pathogen-induced intestinal inflammation (**Figure 2**). For example, *S. Typhimurium* effector SopF regulated PANoptosis in IEC to attenuate intestinal inflammation. Specifically, SopF inactivated caspase-8 through the PDK1-RSK signaling, thereby inhibiting apoptosis and pyroptosis of IEC with the promotion of necroptosis. Thus, SopF restricted the dislodging of IECs to promote bacterial dissemination, which exacerbates systemic infection (60). In addition, ventilator-induced lung injury (VILI) substantially promoted the expression of caspase-3, N-GSDMD and p-RIPK3 in the gut due to systemic cytokines, suggesting that PANoptosis involved in VILI-induced gut injury and inflammation in the mice (61).

Recently, PANoptosis has been implicated in the onset and progress of IBD. Based on multiple transcriptome profiles of intestinal mucosal biopsies from the GEO database, bioinformatics analysis identified that four pivotal PANoptosis-related gene (ZBP1, AIM2, CASP1/8) were significantly activated in UC patients, which regulated specific immune cells and interacted with key signaling pathways contributing to the pathogenesis of UC. These findings were validated in the DSS-induced mouse colitis model (62–64). Another comprehensive study combining bioinformatics, machine learning, and experimentation confirmed that PANoptosis played an undeniable role in CD by regulating the immune system and interacting with CD-related genes (65). The abnormal activation of ZBP1 caused embryonic lethality and intestinal cell death. In mouse models, the knockdown of key sensor molecules of PANoptosis has been shown to rescue the death fate of innate immune-induced epithelial cells (66). Researches indicated the crucial role of IFN-γ in inducing the intestinal epithelial barriers (67). The result obtained from human intestinal organoid (enteorid) model has shown that PANoptosis is the major mechanism of IFN-γ-induced IEC damage. Furthermore, bulk and single-cell RNA sequencing (RNA-seq) found that PCD-associated gene expression was upregulated in enterocytes and goblet cells, but not in intstinal stem cells and Paneth cells (68). This finding demonstrates IFN-γ-induced intestinal epithelial cell type-specific PANoptosis. Mechanistically, NLRC5 could function as an innate immune sensor in inflammatory conditions and interact with NLRP12 to form a PANoptosome in response to NAD^+^ depletion. Thus, deletion of NLRC5 protect mice from colitis (69). However, this study did not illuminate whether NLRC5-mediated PANoptosome formation bears intestinal epithelial cell type specificity.

IFN regulatory factor 1 (IRF1) is a transcription factor of the PANoptosome sensors ZBP1 and NLRP3, and knocking out IRF1 will reduce the expression of PANoptosome core molecules such as NLRP3, CSAP1/3/8, and MLKL in the innate immune response (70, 71). In a UC mouse model, the knockdown of IRF1 also significantly inhibited the expression of colonic apoptosis, pyroptosis, and necroptosis executioner proteins CASP3/7, GSDMD, and MLKL, thus inhibiting PANoptosis (72). Colitis-associated cancer (CAC) is the most serious complication of inflammatory bowel disease, which is driven by long-term inflammatory damage. In the case of CAC, loss of IRF1 suppressed PANoptosis of colon, thereby increasing the susceptibility of Irf1^-/-^ mice to CRC (72). Hence, IRF1 functions as a key upstream mediator of PANoptosis, which could potentially be a therapeutic target for IBD. More in-deep and systematic investigation is warranted to uncover the substantial insights into the essential role of IRF1 in the PANoptosis process of IEC, which is beneficial for a momentously advanced understanding of the etiology and pathogenesis of IBD.

### 4.2 Potential nature compounds targeting IEC PANoptosis in IBD

It is widely acknowledged that in pathological contexts, three distinct modalities of cell demise can coexist, exhibiting overlapping mechanisms and functioning as complementary death strategies. For instance, the inhibition of caspase-8 can effectively mitigate the process of apoptosis; however, this intervention may precipitate alternative forms of cell death, such as necroptosis or pyroptosis. Notably, PANapoptosis encompasses all three types of cell death, and thus, targeting PANapoptosis presents a strategic approach to concurrently inhibit these three distinct modes of cell demise.

It’s well-established that natural products stemmed from multifarious medicinal plants, vegetables and fruits orchestrate different cell death modalities for IBD treatment with lower costs, flexible dosage adjustments, fewer side effects and long-term application (73, 74). However, no reports delineate natural products regulating PANoptosis of IEC for IBD treatment. Nevertheless, numerous natural products are currently being investigated as PCD regulators for IBD therapy. Natural compounds that modulate both apoptosis and necroptosis or apoptosis and pyroptosis in IEC are listed in **Table 1**, setting a precursor for research into PANoptosis of IEC in IBD.

**Table 1:** Compounds that induce cell.

Natural compounds	Cell death	*In Vivo*/Vitro	Cell lines/Animals	Mechanisms	References
HuanglianGanjiang Tang (HGT) and the compound berberine	Necroptosis	*In Vitro*	Mice with DSS-induced colitis	Activating vitamin D receptor (VDR) signaling pathway	(75)
berberine	Apoptosis	*In Vivo* and *In Vitro*	Caco-2 Cells/ Mice with DSS-induced colitis	Lowering JNK phosphorylation	(76, 77)
	Pyroptosis	*In Vivo* and *In Vitro*	Caco-2 Cells/ Mice with DSS-induced colitis	Activating the Wnt/β-catenin pathway via modulating the miR-103a-3p/BRD4 axis	(78)
L. edodes polysaccharides	Necroptosis	*In Vivo* and *In Vitro*	Caco-2 Cells/ Mice with DSS-induced colitis	Inhibiting pMLKL-mediated necroptosis	(79)
	Pyroptosis	*In Vitro*	Human umbilical vein endothelial cells	Regulating the LncRNA MALAT1/miR-199b/mTOR axis and the NLRP3/Caspase-1/GSDMD pathway	(80)
Traditional herbal formula Wu-Mei-Wan (WMW) and the compound ginsenoside Rb1	Necroptosis	*In Vivo*	Mice with TNBS-induced colitis	Promoting RIPK3 O-GlcNAcylation and suppressing the binding of RIPK3 and MLKL	(81)
Ginsenoside Rb1	Apoptosis	*In Vivo* and *In Vitro*	IEC-6 Cells/ Mice with DSS- and TNBS-induced colitis	Activating Hrd1 signaling pathway	(82)
	Pyroptosis	*In Vitro*	Non-IEC (astrocytes, hepatocytes and renal cells)	Activating mitophagy	(83–85)
Cucurbitacin E	PANoptosis	*In Vitro*	Non-IEC	Regulating ZBP1	(86)

HuanglianGanjiang Tang (HGT) is a renowned prescription of traditional Chinese medicine (TCM). HGT obstructed necroptosis in IEC by activating vitamin D receptor (VDR) signaling pathway, thereby attenuating DSS-induced colitis. Furthermore, molecular docking analysis has successfully proved the binding affinity of the five compounds to VDR, including berberine, phellodendrine, 6-Gingerol, ferulic acid and citric acid (75). Previous studies uncovered that berberine could evidently lessen cytokine-induced Caco-2 apoptosis *in vitro* by lowering JNK phosphorylation, thus in turn promoting the recovery of colon epithelium in DSS-treated mice (76, 77). Recently, a research found that berberine weakened colitis-induced pyroptosis and intestinal mucosal barrier defects by activating the Wnt/β-catenin pathway via modulating the miR-103a-3p/BRD4 axis (78).

The natural compound polysaccharides from the edible mushrooms *Lentinus edodes* showed therapeutic properties on DSS-induced colitis. The carbohydrate-rich component of *L. edodes* polysaccharides suppressed TNF-induced cell death of Caco-2 cells in a dose-dependent manner by inhibiting pMLKL-mediated necroptotic cell death, thus counteracting DSS-induced colitis in mice (79). This study also revealed that L. edodes polysaccharides prevented apoptotic cell death in Caco-2 cells (79). The effect of *L. edodes* polysaccharides on pyroptosis is reported in non-IEC. In human umbilical vein endothelial cells, *L. edodes* polysaccharides dampened advanced glycation end products (AGEs)-induced pyroptosis via regulating the LncRNA MALAT1/miR-199b/mTOR axis and the NLRP3/Caspase-1/GSDMD pathway (80).

Traditional herbal formula Wu-Mei-Wan (WMW) could augment colonic O-GlcNAc transferase (OGT) activity and inhibit O-GlcNAcase (OGA) activity, which may be regulated by the compounds hesperidin, coptisine and ginsenoside Rb1 found in WMW. As a result, WMW could prevent necroptosis through promoting RIPK3 O-GlcNAcylation and suppressing the binding of RIPK3 and MLKL, ultimately alleviating TNBS-induced colitis in mice (81). A previous study demonstrated that ginsenoside Rb1 abated LPS-induced apoptosis via activating Hrd1 signaling pathway in intestinal cell line IEC-6, thus alleviating colitis symptoms in DSS- and TNBS-treated mice (82). Additionally, ginsenoside Rb1 diminished pyroptosis by activating mitophagy in non-IEC, such as astrocytes, hepatocytes and renal cells (83–85). Importantly, forthcoming research endeavors about the effect of ginsenoside Rb1 on the IEC and colitis are necessary.

Cucurbitacin E (CurE), a natural product extracted from plants in the *Cucurbitaceae* family. In the adrenocortical carcinoma cells, is a CDK1 inhibitor. CDK1 regulated the PANoptosis of adrenocortical carcinoma cells through binding with the PANoptosome in a ZBP1−dependent way (86). Machine learning and integrated bioinformatics identified possible hub genes (AURKB, CDK1, and CCNA2) between bladder cancer and inflammatory bowel disease (87). Further investigation is warranted to confirm whether cucurbitacin E regulates PANoptosis in a ZBP1−dependent way in the context of IBD specifically.

In a word, berberine, *L. edodes* polysaccharides, ginsenoside Rb1 and cucurbitacin E are potential natural compounds that regulate PANoptosis of IEC for the treatment of IBD. These evidence pave the way for future pharmacological research.

## 5 Conclusion and discussion

Over the past decade, a wealth of evidence has established the basic knowledge on apoptosis, necroptosis and pyroptosis and their roles in the pathogenesis of IBD. This review followed with interest in the current understanding of the crosstalk between these cell deaths modalities in IEC, particularly in the context of inflammatory bowel disease. However, IEC constitute a heterogeneous population along the gut, embodying absorptive cells, goblet cells, enteroendocrine cells, Paneth cells, M cells, cup cells, and Tuft cells. The significance of location-specific and cell type-specific cell death along the length of the intestine has been highlighted by various transgenic mouse models. Further investigation is needful to unravel the cell death and their crosstalk at single-cell resolution in different IEC types from the intestine, which will potentially illuminate the underlying nature of IBD.

Within the landscape about the crosstalk between apoptosis, necroptosis and pyroptosis in IEC, this review further provided an overview about the IEC PANoptosis in the context of IBD. However, the body of literatures addressing this topic is scant. The study of PANoptosis in IBD remains limited to in preliminary experimental phases. As such, there is an imperative need to probe more intricately into the underlying mechanisms governing PANoptosis and the upstream modulators in IEC through both foundational research and clinical trials. This endeavor will undoubtedly foster the emergence of innovative and more efficacious treatment strategies for IBD.

Given the well-established role of natural products in the prevention and treatment of IBD, coupled with their minimal adverse effects, this review also highlights potential nature compounds targeting IEC PANoptosis for IBD treatment based on the limited literatures. This merits further attention and contemplation. We hope to provide a solid groundwork for researchers in this field to explore the potential drugs for IBD treatment in the foreseeable future.
